# The Effects of a Dietary Supplement (PediaFlù) Plus Standard of Care in Children With Acute Tonsillopharyngitis/Rhinopharyngitis: Protocol for a Randomized Controlled Trial

**DOI:** 10.2196/53703

**Published:** 2024-05-31

**Authors:** Fabio Cardinale, Dionisio Franco Barattini, Federica Sbrocca, Alessandro Centi, Greta Giuntini, Maria Morariu Bordea, Dorina Herteg, Serban Rosu, Cristian Radu Matei

**Affiliations:** 1 Complex Operating Unit Paediatrics Giovanni XXIII Paediatric Hospital University of Bari Bari Italy; 2 Opera Contract Research Organization, a Tigermed company Timisoara Romania; 3 Pediatrica Srl Livorno Italy; 4 Cabinet Medical Medicina de Familie Dr Morariu Bordea Timisoara Romania; 5 Cabinet Medical Dr Herteg Timisoara Romania; 6 University of Medicine and Pharmacy Victor Babes Timisoara Romania; 7 Cabinet Medical Dr Matei Otelu Rosu Romania

**Keywords:** dietary supplements, tonsillitis, pharyngitis, nasopharyngitis, Pelargonium, propolis, zinc, severity score

## Abstract

**Background:**

A dietary supplement containing *Pelargonium sidoides* extract, propolis, zinc, and honey has been recently developed and proven to be an effective adjuvant in clinical practice for seasonal diseases and the treatment of respiratory tract disorders.

**Objective:**

This trial aims to verify the efficacy of the tested dietary supplement in a pediatric population with acute tonsillopharyngitis/rhinopharyngitis (ATR).

**Methods:**

The trial includes children aged between 3 and 10 years with ATR ≤48 h, a negative rapid test for beta-hemolytic streptococcus or culture identification of nasal and/or pharyngeal exudates, and SARS-CoV-2 infection. The dietary supplement tested is an oral solution already on the market based on Pelagon P-70 (equivalent to *Pelargonium sidoides* d.e. 133.3 mg/100 ml), propolis, zinc, and honey. The product is administered at 5 ml 3 times a day for 6 days for children younger than 6 years and 10 ml 3 times a day for 6 days for children older than 6 years. The study design is open label, randomized, and controlled, with the tested dietary supplement plus standard of care (SoC) versus SoC alone. Patients are enrolled from 3 sites in Romania. The change in Tonsillitis Severity Score and number of treatment failures (using ibuprofen or high-dose paracetamol as rescue medication) are the primary end points. Based on the Tonsillitis Severity Score and the 2-sample comparison of the means formula with a 5% significance level, 80% power, and a minimally clinically important difference of 2 (SD 3.85) points, 120 patients are required. To account for potential screening failures and dropouts, we need to screen a population of approximately 150 children.

**Results:**

Patient enrollment began on June 3, 2021 (first patient’s first visit), and ended on August 12, 2021 (last patient’s last visit). The data collection period was from June 3, 2021, to September 16, 2021. The study was funded in February 2023. Data analysis is currently ongoing (April 2024). We expect the results to be published in a peer-reviewed clinical journal in the third quarter of 2024 and presented at scientific meetings in the last quarter of 2024.

**Conclusions:**

The data from this trial may help identify new adjuvant treatments for children with ATR when streptococcal infection is excluded by a negative rapid test, thereby avoiding unnecessary antibiotic administration.

**Trial Registration:**

ClinicalTrials.gov NCT04899401 https://clinicaltrials.gov/study/NCT04899401

**International Registered Report Identifier (IRRID):**

DERR1-10.2196/53703

## Introduction

### Background

Acute tonsillopharyngitis/rhinopharyngitis (ATR) is a leading cause of pediatric consultations among children and adolescents [1]. Viruses are the primary causative agents of ATR [2], whereas bacterial infection is the cause in only a small percentage (approximately 30%) of cases [2,3]. ATR diagnosis primarily relies on clinical evaluation and is confirmed using cell culture or rapid antigen tests. The predominant symptoms of virus-induced ATR include sore throat, dysphagia, cervical lymphadenopathy, and fever. Antibiotic treatment is exclusively recommended for bacterial tonsillopharyngitis as it encourages antibiotic resistance and can cause adverse reactions [4,5]. Therefore, the focus is on symptom control and acceleration of recovery through analgesia, hydration, and rest [6], with the necessity for new treatment approaches coming to light.

*Pelargonium sidoides* has proven efficacy in managing respiratory tract disorders [7-13]. A long tradition of use and literature also supports the use of zinc, propolis, and honey to prevent seasonal diseases and as adjuvants during the acute phase of the common cold [14,15]. These findings underscore the potential of these natural remedies for treating colds. Therapeutic zinc therapy may be effective in inhibiting viral replication and reducing the duration of symptoms associated with the common cold [16]. The flavonoid content of propolis contributes to its antibacterial, antiviral, and anti-inflammatory properties [17]. A systematic review and meta-analysis demonstrated that honey is more effective than usual care in improving symptoms of upper respiratory tract infections (URTI) [18].

### Rationale

A recent review of the medical literature indicates that the median incidence of streptococcal pharyngitis is 1764 per 100,000 children [19]. Considering the small subset of patients who experience streptococcal infections and that these cases rarely develop late complications, such as rheumatic fever, the widespread prescription of antibiotics for sore throats has become increasingly criticized. These opinions are supported by the Committee on Infectious Diseases of the American Academy of Pediatrics [20], which recommends antibiotic therapy only for children with pharyngitis clearly caused by group A streptococcus. Therefore, new therapeutic strategies are needed for the first-line treatment of acute respiratory tract infections beyond the strict indication for antibiotic treatment, in addition to the purely symptomatic treatment recommended in various guidelines.

Recently, Pediatrica Srl (Livorno, Italy) developed PediaFlù, a dietary supplement for the pediatric population (DSPP) specifically designed for the well-being of the respiratory tract, containing an extract of *Pelargonium sidoides*, propolis, zinc, and honey. Its efficacy has been proven on a bibliographic basis as required for dietary supplements.

Its efficacy has been proven on a bibliographic basis, as is required for dietary supplements. The product is currently marketed as an adjuvant for seasonal illnesses and could now be evaluated as a potential add-on to the standard of care (SoC) for children with ATR.

### Objectives

This clinical trial aims to verify and confirm the positive results observed in pediatric clinical practice. The study is designed to evaluate the effectiveness and safety of this DSPP in combination with SoC for 6 days compared to SoC alone in children with ATR. The visual abstract is presented in Multimedia Appendix 1.

## Methods

### Overview

The project team is composed of the scientific supervisor of the study, who prepared the outline of the study protocol, and 3 investigators in Timișoara (Romania), coordinated by the Academic Research Organization Department of Clinical Trials of the University of Medicine and Pharmacy Victor Babes.

The team outlined the following points: (1) ATR is diagnosed through a clinical evaluation of symptoms, including sore throat and catarrhal angina, and complaints lasting ≤48 h; (2) a negative result from a rapid test for group A beta-hemolytic streptococcus (GABHS) or culture, and the identification of nasal and/or pharyngeal exudates, along with a negative SARS-CoV-2 test are mandatory; (3) the Tonsillitis Symptom Score (TSS) should be greater than or equal to 8 points; and (4) patients should undergo follow-up for 6 days after the onset of ATR.

The study team submitted the study protocol to Pediatrica Srl. The company provided the study team with dietary supplements and a partial grant to conduct a study on ATR among children. The agreement between the parties specified that the sponsor would have no role in the study design and planning or the collection, analysis, and interpretation of data in a future manuscript reporting the results.

Finally, the team selected Opera CRO, a Tigermed company (Timisoara, Romania) as the contract research organization (CRO) responsible for the logistics and management of the study (protocol submission, project management, site monitoring, data management, and statistical analysis).

### Ethical Considerations

A total of 3 centers in Romania are participating in this study. Following approval by the National Agency for Medicines and Medical Devices (Agentia Nationala a Medicamentului si a Dispozitivelor Medicale), the protocol was approved by the local ethics committee (EC) Cabinet Medical Dr Cristian Radu Matei in Oțelu Roșu (Romania) on April 23, 2021. The remaining 2 Romanian ECs in Timisoara approved this study a few days later. Cabinet Medical Medicina de Familie Dr. Morariu Bordea EC granted approval on April 27, 2021, whereas the Cabinet Medical Dr. Herteg Dorina EC provided approval on the same day. The EC approvals are reported in Multimedia Appendix 2.

Informed consent from parents of each potentially eligible child followed the International Council for Harmonization-Good Clinical Practice (ICH-GCP) and Declaration of Helsinki guidelines. A patient information sheet, approved by the local EC and written in plain Romanian, is provided that outlines the study purpose, procedures, and the food supplement’s nature and safety profile. It also outlines visit frequency, procedures, and potential outcomes. This document ensures that the parents understand the risks, benefits, and discomforts. The sheet is regularly updated with product and study information that could be available in the future. Parents are informed about the voluntary nature of their child's participation in the study, emphasizing their right to withdraw at any time during medical care without repercussions. They are notified that no compensation is offered for participation and that insurance coverage is provided by the sponsor in case of trial-related injuries. Additionally, parents are made aware that their children's medical records may be accessed by the sponsor, CRO personnel, and regulatory authorities as per relevant laws and regulations, with personal information maintained in a confidential database.

Informed consent is obtained after allowing participants sufficient time to review the information and ask questions. The written consent process entails both the parent and investigator manually writing and signing their names on 2 copies of the consent form. One copy of the signed consent form and 1 copy of the signed informed consent form (ICF) are provided to the parents, while the second copy of the original signed forms is retained in the on-site study file. The study adheres to current General Data Protection Regulations (GDPR). Specific procedures for data collection, anonymization, and the possibility of data sharing with other researchers in aggregated and anonymous form are outlined in a dedicated document (GDPR form) written in the local language. This document is provided to parents for approval and signature before the study commences. The physician in charge of each center bears responsibility for managing patient data at the clinical site in compliance with the GDPR.

The study is registered at Clinical Trials.gov (NCT04899401).

### Study Design

This study is a multicenter, controlled, open-label, randomized parallel-group superiority trial. The study formulates a superiority hypothesis to assess whether symptoms in patients with ATR can be improved by a 6-day course of DSPP plus SoC compared with SoC alone.

Screened patients who meet all eligibility criteria for the study are randomly assigned to either study arm, SoC plus DSPP or SoC alone, receive sufficient products to complete the study, and are instructed on how to adhere to the assigned treatment.

Randomization is conducted using an internet-based platform provided by the CRO, which is available 24 hours a day.

Patients are allocated in a 1:1 ratio to the control group receiving SoC or the interventional group receiving DSPP plus SoC.

The allocation is carried out by a noninvolved staff member and the patients. The patients’ parents are not blinded to the process.

### Study Setting and Patient Recruitment

The 3 study centers were selected for their extensive experience in ATR treatment in children and their wide catchment area for patients with this condition. The coordinating center, Cabinet Medical Medicina de Familie Dr Maria Morariu Bordea, is situated in Timișoara (Romania), while the other 2 centers are satellite sites: Cabinet Medical Dr Dorina Herteg in Timișoara and Cabinet Medical Dr Cristian Radu Matei in Otelu Rosu (Table 1). At each center, a principal investigator is responsible for the identification, recruitment, data collection, and completion of case report forms (CRFs), as well as for checking patients' compliance with the protocol during the study period.

Only children who were accompanied by their parents or caregivers to the participating clinical sites during the study period were enrolled in the study. No flyers, social media, or other advertising methods were used to invite patients to participate in the study.

The time required to define the study protocol and prepare relevant documents for study submission (informed consent, patient information sheet, and CRF) was longer than originally planned. As a result, EC approval was received in April, and the first patient enrollment at the 3 sites occurred between June 3 and June 21, 2021, during the summer period. Even if viral infections are less prevalent during this period, the investigators were able to complete enrollment in only 3 months, with the last patient visit occurring on September 16, 2021

**Table 1:** Centers involved in the study.

Center type	Investigators^a^ (study coauthors)	Hospital	Town	FPFV^b^ and LPLV^c^
Coordinator center	MMB	Cabinet Medical Medicina de Familie Dr Maria Morariu Bordea	Timisoara	June 21, 2021, and September 16, 2021
Satellite center	DH	Cabinet Medical Dr Dorina Herteg	Timisoara	June 3, 2021, and September 16, 2021
Satellite center	CRM	Cabinet Medical Dr Cristian Radu Matei	Otelu Rosu	June 7, 2021, and September 16, 2021

^a^The scientific supervisor of the study is author FC.

^b^FPFV: first patient’s first visit.

^c^LPLV: last patient’s last visit.

### Inclusion and Exclusion Criteria

The inclusion and exclusion criteria for this study are outlined in Textbox 1.

**Study inclusion and exclusion criteria.**

**Inclusion criteria**

- Male and female children aged 3 to 10 years
- Acute tonsillopharyngitis/rhinopharyngitis (ATR; sore throat, catarrhal angina), duration of symptoms ≤48 h
- Negative with rapid test for group A beta-hemolytic streptococcus (GABHS) or culture and identification of nasal and/or pharyngeal exudates; negative for SARS-CoV-2 infection
- Tonsillitis Symptom Score (TSS) ≥8 points
- Both parents are willing to provide written informed consent prior to participation in the clinical trial.
- Children older than 6 years are able the ability and willingness to provide written informed consent.

**Exclusion criteria**

- Evidence of lacunar or follicular angina
- More than 2 past episodes of tonsillitis in the previous 12 months
- Mandatory indication for antibiotic therapy (eg, abscess, septic tonsillitis, status postrheumatic fever, poststreptococcal glomerulonephritis, and minor Sydenham chorea)
- Treatment with antibiotics within 4 months prior to study enrollment
- Hemorrhagic diathesis increases and chronic illnesses (eg, severe heart, kidney, or liver disease, and primary or secondary immunodeficiencies)
- Close contact history with individuals infected with SARS-CoV-2 within 10 days before showing symptoms
- Known or suspected allergic reactions to any study medication
- Concomitant therapy that may affect study results or have known interactions with study medications (such as coumarin derivatives)
- Participation in another clinical trial within 3 months before enrollment

### Intervention

#### Tested Product

The DSPP tested is a dietary supplement already on the market; its composition is based on Pelagon P-7 (equivalent to *Pelargonium sidoides* d.e. 133.3 mg/100 ml), PropolNext Plus (equivalent to propolis d.e. 7.7 mg/100 ml), zinc (13.3 mg/100 ml), and honey (5.5 g/100 ml). DSPP is supplied as an oral solution, and the instructions for use provide detailed information on its dosage, at 5 ml 3 times a day for children under 6 years for 6 days and 10 ml 3 times a day for children over 6 years of age for 6 days (oral administration for both age groups).

*Pelargonium sidoides* [11] has demonstrated its efficacy in standard clinical practice and medical literature. A double-blind, placebo-controlled clinical trial was conducted in 124 pediatric patients (60 in the active ingredient group and 64 in the placebo group) with acute nonstreptococcal tonsillopharyngitis, wherein an extract from the roots of *Pelargonium sidoides* was administered for 6 days in comparison with a placebo. The primary efficacy variable was the change in the total TSS from baseline on day 4. Treatment with the product was shown to reduce the severity of symptoms and shorten the duration of illness compared with placebo *(P*<.001) [12]. In addition, *Pelargonium sidoides* extract is effective and well-tolerated in the treatment of acute bronchitis in children and adolescents outside strict antibiotic indications [10].

For safety reasons, the investigator was able to stop the administration of DSPP at any time and prescribe other therapies necessary for patient health.

#### Outlining the SoC

In this study, all patients are treated according to the SoC: both children assigned to the control group (SoC only) and those assigned to an intervention group (DSPP plus SoC) for ATR. The products and treatments administered are outlined in Textbox 2.

**The products and treatments administered as part of the standard of care (SoC).**

- Nasopharyngeal lavage by hydration with drinking fluids to support the excretion of body fluids, aspiration of secretions, NaCl solution for nasal irrigation, nasal sprays with seawater, and nasal spray with active ingredients (to be used only when indicated by the physician)
- Throat sprays with benzydamine hydrochloride (Tantum Verde, CSC Pharmaceuticals, 0.15%), pediatric use according to the package insert (each spray corresponds to 0.17 ml of solution), for 6 days:
- Children (under 6 years of age): 1 spray per 4 kg of body weight, up to a maximum of 4 sprays at one time, 2 to 6 times daily
- Children (6 to 12 years): 4 sprays 2 to 6 times daily
- Paracetamol (acetaminophen) (Panadol, GlaxoSmithKline, 120 mg/5 ml): as antipyretic (fever is defined as body temperature >38.5 °C), as needed, 10 mg/kg/dose, every 6 to 8 hours, according to the leaflet; maximum dosage 30 mg/kg/day

#### Rescue Medicine and Interventions Not Permitted

Ibuprofen and paracetamol are the only treatments allowed in cases of need as rescue medicines to relieve disabling symptoms of ATR, when needed, as follows: (1) ibuprofen (Algin Baby, 100 mg/5 ml) per os; (2) high dose (>30 mg/kg/day) paracetamol (Panadol, 120 mg/5 ml) per os. Treatment failure was defined as the use of rescue medication.

Coumarin-based products and antibiotics are not permitted during the study period.

#### Potential Risks

The potential risks to children involved in the study are related to the administration of oral DSPP and rescue medicine (paracetamol). In this section, we do not evaluate the potential risks of SoC because this treatment should be administered only for the benefit of the patient and independently of this interventional clinical trial.

In addition to its decades-long presence on the international market, the efficacy and safety of *Pelargonium sidoides* extract in children’s respiratory pathologies have been widely demonstrated in several placebo-controlled clinical trials and open clinical studies. A recent systematic review [21] evaluated the safety profile of *Pelargonium sidoides* extract based on 29 clinical trials and postmarketing surveillance studies completed by February 2010. Among all the studies, 11 enrolled only children, 14 enrolled only adults, and 4 enrolled both children and adults. Across all studies, a total of 10,026 patients and 31 healthy participants were included. Of these, 8005 individuals were exposed to EPs 7630, 1883 to placebo, 139 to the comparator acetylcysteine (bronchitis), and 30 to symptomatic therapy not otherwise specified. In total, 3939/10,026 (39.3%) patients were infants, children, or adolescents up to the age of 18 years, among whom 3243 were exposed to *Pelargonium sidoides* extract and 527 to placebo. No serious adverse drug reaction was reported in this large patient population, confirming the *Pelargonium sidoides* extract’s optimal safety profile.

Given the limitations of the use of paracetamol (occasional use only, with restrictions on dosage and duration, and exclusion of individuals with renal or hepatic impairment), the associated risk is expected to be minimal. However, the potential risks include hypersensitivity reactions (paresthesia or pruritus transient rash), thrombocytopenia (generally asymptomatic, rarely bleeding or bruising, blood in urine and feces, and black and soft stools), dermatitis, agranulocytosis, oligoanuria, renal colic, and hepatic impairment.

The allowed dosage of ibuprofen as rescue medication in this trial is very low. In addition, prolonged treatments are not permitted, and preexisting cardiovascular, renal, and hepatic impairments are in the exclusion criteria. The remaining possible risks of nonsteroidal anti-inflammatory drugs (NSAIDs) are typical gastrointestinal side effects like bleeding, ulceration, abdominal pain, diarrhea, vomiting urinary tract side effects (cloudy urine, oliguria), hypersensitivity reactions (pruritus, itching skin), unusual bleeding, and weight gain.

### Statistical Methods Analysis

#### Trial Hypothesis or Pass/Fail Criteria

The primary hypothesis is that the minimal clinical difference between the test group (DSPP plus SoC) and the control group (SoC only) will result in a 2-point decrease in mean TSS after 6 days of treatment.

The null hypothesis of this study is that treatment with DSPP plus SoC will not significantly improve the symptoms of the treated patients compared to the control group (treated with SoC alone).

A greater improvement in TSS in the DSPP-treated group from baseline to day 6 of treatment supports the alternative hypothesis and demonstrates the efficacy of the supplement. The overall type I error rate remains at 5%.

#### Sample Size Calculation

The sample size is calculated based on the primary outcome of the TSS and the results of a similar study [12].

**Figure 1:**
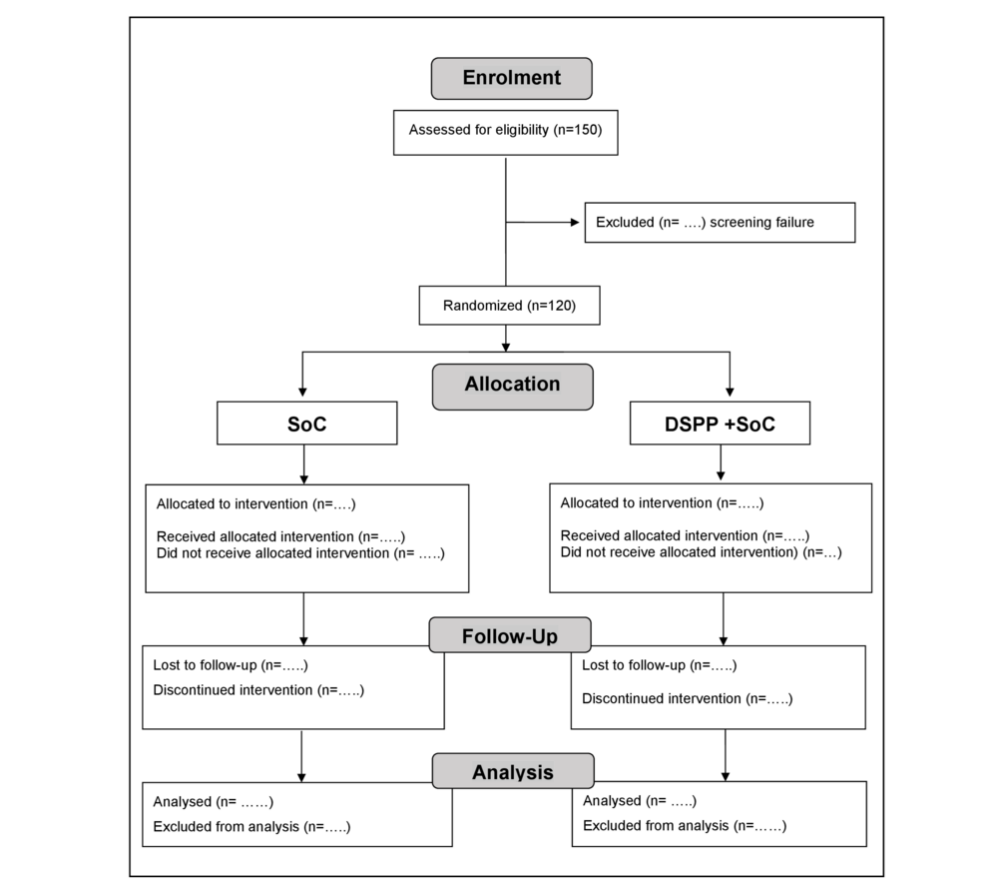
CONSORT (Consolidated Standards of Reporting Trials) 2010 flow diagram. DSPP: dietary supplement; SoC: standard of care.

Based on the sample size formula for comparing 2 means (2-sample) at a 5% significance level, 80% power, and a minimum clinically important difference of 2 (SD 3.85 points), 120 patients are required. To obtain 120 evaluable participants, approximately 150 will be screened (including potential screening failures and estimated dropout patients). Figure 1 shows the CONSORT (Consolidated Standards of Reporting Trials) diagram.

### Statistical Analyses

All statistical analyses are performed using R statistical software (version 4.1.0; R Foundation for Statistical Computing). The final analysis will be performed after all patients have completed the study, all queries have been resolved, and the database has been locked.

The overall type I error rate is maintained at 5%. All tests are 2-sided. Data from unscheduled visits are excluded from the analyses.

Statistical analyses are performed for all patients who completed the study without protocol deviation, which is considered to affect the assessment of key variables. The quality and completeness of the collected data are assessed before data analysis. If a patient has missing information for 1 or more variables, the missing data are not replaced, even after the queries are resolved. If a patient violates the inclusion/exclusion criteria, the corresponding data are excluded from analysis.

Quantitative (ie, demographic) variables, if normally distributed, are described by mean and SD; nonnormally distributed variables are described by median and IQR. The Student *t* test and Mann-Whitney U test are used to perform comparative analyses according to the distribution of these variables. Factorial analysis of variance can also be used to evaluate any interactions between quantitative variables and linear progression models to relate possible confounding biases to the independent variables.

Categorical variables are described using frequencies and percentages, and comparative analyses are performed using the chi-square test.

### Evaluation Outcomes

#### Primary Outcomes

The primary efficacy outcomes investigated are the change in TSS and the number of treatment failures.

The TSS is composed of 5 subscores that reflect the nature of acute non-GABHS tonsillopharyngitis as a viral-induced inflammatory URTI: difficulty swallowing, sore throat, increased salivation, pharyngeal erythema, and fever. At the visits, each of the symptoms of difficulty swallowing, sore throat, increased salivation, and pharyngeal erythema is rated by the investigator as severe (3), moderate (2), mild (1), or absent (0). Finally, a fever score is added with the categories <37.5 °C=0, 37.5 °C to <38.5 °C=1, 38.5 °C to <39.5 °C=2, and ≥39.5 °C=3, resulting in a possible TSS range of 0 to 15. The results are compared in terms of absolute change in score from baseline to the final visit, between groups, and within groups. The TSS (total score and subscores) is assessed and scored at each visit.

The number of treatment failures is calculated by comparing the administration of rescue medication (paracetamol >30 mg/kg/dose or ibuprofen 100 mg/5 ml) between the 2 groups.

The primary safety outcome is the incidence of adverse events (AEs) and serious adverse events (SAEs) during the study.

#### Secondary Outcomes

The following outcomes are collected:

First, the Investigator Global Assessment of Efficacy (IGAE). It uses a 4-point scale ranging from 1 (excellent to 4 (poor), and the comparison is between groups at the end of the study.

Second, the Patient Global Assessment of Efficacy (PGAE). The outcome is rated by patients on a 5-point scale, ranging from 1 (very satisfied) to 5 (very unsatisfied). A comparison is made between groups at the end of the trial.

Third, the Investigator Global Assessment of Safety (IGAS). This assessment is made by the investigators using a 4-point scale ranging from 1 (very good safety) to 4 (poor safety). A comparison is made between groups at the end of the trial.

Compliance is also assessed between the groups at the end of the study.

### Study Procedures and Visits

Prior to each study procedure, each patient and their parent or legal guardian are informed of the purpose and nature of the study, as well as the potential risks and benefits. They are also asked to give their consent by signing an ICF.

The study timeline is presented in Table 2.

**Figure 2:**
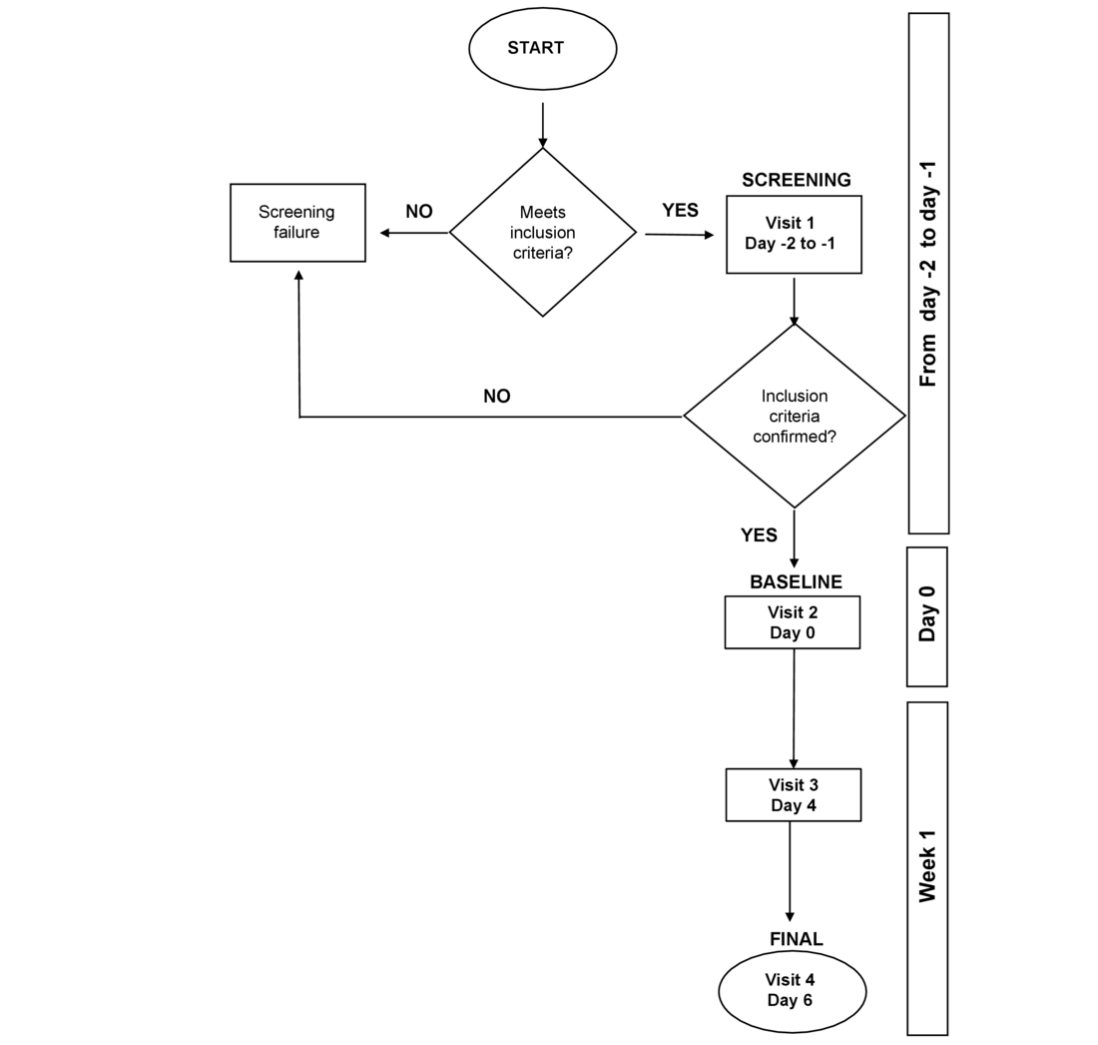
Flow chart of the study.

A total of 4 visits are planned for the study (Figure 2). At each visit, an eligibility assessment (or confirmation) and physical examination (including disease assessment and TSS score evaluation) are performed, and concomitant medications and AE/SAE are assessed and recorded.

In addition, at the screening visit (visit 1; day –2 to day –1), GABHS and SAR-CoV-2 screening is performed, medical history and demographics are recorded, and parents or legal guardians of the eligible children are asked to sign the ICF. At the baseline visit (visit 2; day 0), each participant’s baseline characteristics are recorded, and study products and patient diaries are supplied. The diary is reviewed at the interim visit (visit 3; day 4 of enrollment). At the end-of-study visit (visit 4; day 6 of enrollment), final assessments, symptom and compliance assessments are performed, and the study products and diaries are collected.

**Table 2:** Timeline of the study

Time point	Visit 1 (day –2 to –1)	Visit 2 (day 0)	Visit 3 (day 4)	Visit 4 (day 6)
Informed consent	✓			
Inclusion criteria	✓			
Exclusion criteria	✓	✓	✓	✓
Demographics and medical history	✓			
Physical examination	✓	✓	✓	✓
Disease assessment	✓	✓	✓	✓
Rapid test for GABHS^a^ or nasal and/or pharyngeal exudate culture plus SARS-CoV-2	✓			
Concomitant treatments	✓	✓	✓	✓
TSS^b^	✓	✓	✓	✓
Product delivery		✓		
Product return				✓
Patient diary delivery		✓		
Patient diary verification			✓	
Patient diary return				✓
Product accountability				✓
IGAE^c^				✓
PGAE^d^				✓
IGAS^e^				✓
Adverse events	✓	✓	✓	✓

^a^GABHS: group A beta-hemolytic streptococcus.

^b^TSS: Tonsillitis Symptom Score.

^c^IGAE: Investigator Global Assessment of Efficacy.

^d^PGAE: Patient Global Assessment of Efficacy.

^e^IGAS: Investigator Global Assessment of Safety.

## Results

Patient recruitment started on June 3, 2021 (first patient’s first visit), and ended on August 12, 2021 (last patient’s last visit). The data collection period was from June 3, 2021, to September 16, 2021. The study was funded in February 2023. Data analysis is currently ongoing (April 2024). We expect the results to be published in a peer-reviewed clinical journal in the third quarter of 2024. The results will be also presented at scientific meetings in the last quarter of 2024.

## Discussion

### Comparison With Prior Works

Physicians and pediatricians recognize that the use of antibiotics for viral pharyngitis may be inappropriate, expensive, and contribute to antimicrobial resistance, in addition to causing adverse reactions (ie, rash, abdominal pain, diarrhea, and vomiting) without medical benefit. As noted by several authors, the balance between modest symptom reduction and potential risks of antimicrobial resistance must be recognized [4]. Thus, new strategies are needed, at least when streptococcal infection is excluded by a negative rapid test, as in this study, where only children with ATR for no more than 48 hours and a negative rapid test for GABHS and SARS-CoV-2 infection were included. In addition, to avoid the inclusion of any participants with bacterial diseases, a negative culture of the nasal/pharyngeal exudate was required.

### Limitations of the Study

The main limitation of this study is its open-label and controlled rather than double-blind design. This choice is motivated by logistical and economic reasons.

### Conclusions

When developing this study, we paid particular attention to the selection of the primary outcome. This choice was difficult and laborious; however, in our opinion, it is the most qualified point of our work. The ideal characteristics of the primary outcome had to: (1) be scientifically robust and already used in clinical trials published in the pediatric literature; (2) be proven to be a sensitive tool capable of highlighting the effectiveness of one treatment over another in trials; (3) have a direct relationship with pediatric clinical practice, represented by clinical objectivity and the detection of patients' signs and symptoms; and (4) be aligned with the objective of confirming the positive results observed by many pediatricians administering DSPP with SoC in their clinical practice for children with viral ATR. A reduction in the total TSS score as the main outcome met these needs.

The TSS is a widely used test in the medical literature [22,23]. Recently, the TSS has also been shown to be a sensitive and reliable tool for measuring the overall clinical effectiveness of treatment. It was shown that in 236 children with ATR [24], the marked reduction in TSS scores after antimicrobial treatment in severe and moderate cases indicated the high efficacy of these treatments for diseases with higher severity. In the same study, mild cases showed a decrease in scores regardless of antimicrobial treatment. Thus, using the outcome results in terms of reduction of the total TSS score will reflect the severity of the disease well and be useful for assessing the clinical course and monitoring and evaluating the effectiveness of treatment during the 6 study days. In addition, the TSS subscores are a direct expression of the symptoms that the pediatrician verifies and measures in the child and, therefore, represent the outcome closest to their sensitivity and daily practice. Finally, to avoid compartmentalizing judgment by reducing it to individual clinical symptoms or signs, we considered the global subjective evaluation by the investigator and parents as secondary outcomes.

It must be emphasized that our goal is not only to demonstrate that DSPP associated with SoC will produce a clinical benefit but also that this administration is accompanied by good DSPP compliance and optimal safety, both of which represent the fundamental requirements for a product to be administered in such young children. In this case, our research hypothesis of associating the use of DSPP with SoC may represent a potential therapeutic alternative in the first-line treatment of acute respiratory tract infections beyond the strict indication for antibiotic treatment, in addition to the purely symptomatic treatment recommended by various guidelines. This research hypothesis should also validate the therapeutic approach of thousands of pediatricians who use this product, marketed as an adjuvant for seasonal illnesses, in daily clinical practice. In fact, initial treatment with DSPP may also be helpful between the patient's presentation and the final decision to administer an appropriate antibiotic, providing the benefit of rapid symptomatic improvement regardless of the bacterial or viral etiology of the respiratory illness.

Because we believe that the confirmation of our research hypothesis may be relevant to the therapeutic practice of many pediatricians, we will make a special effort to disseminate the results of our study. Therefore, as soon as we have completed the process of signing the clinical study report, we will upload the results to ClinicalTrials.gov and prepare an article in accordance with the CONSORT statement and in a format that will allow it to be included in a potential systematic review. This manuscript will be submitted for publication in a peer-reviewed journal and presented as a poster at the scientific meeting. It will also be submitted in parallel to a preprint journal.

## Appendix

Multimedia Appendix 1 Visual abstract.

Multimedia Appendix 2 Ethics committees' approvals.

## Abbreviations

AE: adverse event
ATR: acute tonsillopharyngitis/rhinopharyngitis
CONSORT: Consolidated Standards of Reporting Trials
CRF: case report form
CRO: contract research organization
DSPP: dietary supplement for the pediatric population
EC: ethics committee
GABHS: group a beta-hemolytic streptococcus
GDPR: General Data Protection Regulations
ICF: informed consent form
ICH-GCP: International Council for Harmonization Good Clinical Practice
IGAE: Investigator Global Assessment of Efficacy
IGAS: Investigator Global Assessment of Safety
NSAID: nonsteroidal anti-inflammatory drug
PGAE: Patient Global Assessment of Efficacy
SAE: serious adverse event
SoC: standard of care
TSS: Tonsillitis Severity Score
URTI: upper respiratory tract infection

## Data Availability

The full protocol of the study has been deposited at Protocols.io. The data sets analyzed for this study are available from the corresponding author upon request.
