# Supplementary material for: The Effects of a Dietary Supplement (PediaFlù) Plus Standard of Care in Children With Acute Tonsillopharyngitis/Rhinopharyngitis: Protocol for a Randomized Controlled Trial
Source: JMIR Res Protoc. 2024 May 31;13:e53703. doi: 10.2196/53703 (PMC11179036; doi:10.2196/53703)

The Effects of Dietary supplement (PediaFlu®) plus Standard of Care in children with acute Tonsillopharyngitis/ Rhinopharyngitis: Protocol for an Interventional, Randomized, Open-Label, Parallel Group, Controlled Clinical Trial versus Standard of Care Alone

Fabio Cardinale[a]*, Dionisio Franco Barattini[b], Federica Sbrocca[b], Alessandro Centi[c], Greta Giuntini[c], Maria Morariu Bordea[d], Dorina Herțeg[e], Cristian Radu Matei[f]

a: UOC di Pediatria e PS, Azienda Ospedaliero-Universitaria "Policlinico-Giovanni XXIII", Ospedale Pediatrico Giovanni XXIII - Università di Bari
b: Opera CRO, a TIGERMED company, Timișoara, Romania
c: Pediatrica Srl, Livorno, Italy
d: CMMF Dr. Morariu Bordea, Timișoara, Romania
e: CM Dr. Herțeg Dorina, Timișoara, Romania
f: CM Dr. Matei Crisian-Radu, Timișoara, Romania

Conflict of interest

FC, SR, MMB, DH, and CRM declare no conflict of interest.

DFB and FS are employed at Opera CRO, the Contract Research Organization that managed the study

GG and AC are employed at Pediatrica Srl

Funding

Pediatrica Srl (Livorno, Italy) provided grant support for the conduct of the study.

Pediatrica Srl had no role in the study design, data collection, and interpretation, or in the decision to submit the manuscript to medical journals or congresses.

Collaboration

The data monitoring, source data verification, quality assurance, and statistical analysis were performed by the Contract Research Organization Opera CRO (Timisoara, Romania).

Study Design

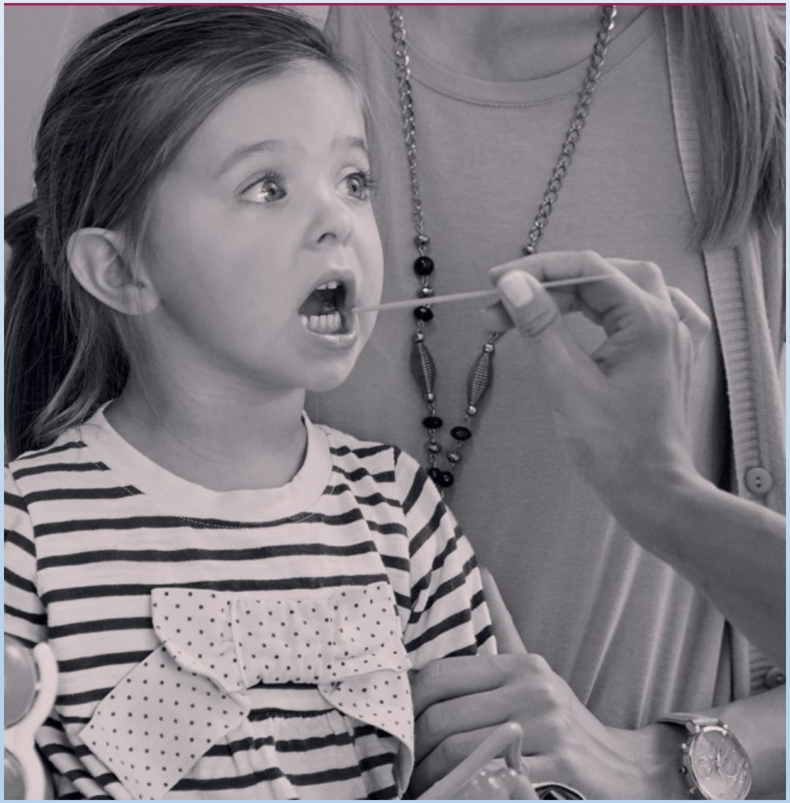

Randomized, open-label, controlled, multicenter trial
with a hypothesis of superiority

2 arms
120 children evaluable in total

3 Clinical Sites located in Romania

Introduction

Acute tonsillopharyngitis (ATP), often referred to as angina catarrhalis in Europe, affects mainly children, adolescent and young adults and represents one of the **most common reason** to consult a family physician.

ATP is highly prevalent, **seasonal** infective disorder characterized by an inflammation of the pharynx and the palatine tonsilis[1].

Children with non-streptococcal tonsillopharyngitis are often **over-treated with antibiotics**[2]**.**

1. *Shaikh N, Leonard E, Martin JM. Prevalence of streptococcal pharyngitis and streptococcal carriage in children: a meta-analysis. Pediatrics 2010 Sep;*
2. *Zanasi A, Lanata L, Saibene F, Fontana G, Dicpinigaitis PV, Venier V, De Blasio F. Prospective study of the efficacy of antibiotics versus antitussive drugs for the management of URTI-related acute cough in children. Multidiscip Respir Med 2016.*

Study population

150 screened children, **3-10 years**.

120 evaluable children, **3-10 years**

3 Pediatric clinics with extensive experience and wide catchment area in children ATR

Absence of pharyngeal exudate and/or Mc Isaac score 0-1 + negative rapid test for β-hemolytic streptococcus and SARS-CoV-2 identification.

The study is approved by the 3 local Ethical Committees (Timisoara, Romania)

Written informed consent was obtained by parents.

Study registered in Clinicaltrials.gov: NCT04899401

Inclusion criteria

- male and female (children 3 - 10 years old);
- acute tonsillopharyngitis/rhinopharyngitis (sore throat, catarrhal angina), duration of complaints ≤ 48 hours;
- negative rapid test for a β-haemolytic streptococcus or nasal and/or pharyngeal exudate culture and identification, and SARS-COV-2 infection;
- tonsillitis symptoms score (TSS) ≥ 8 points;
- written informed consent by both parents;
- willing to provide written informed consent (only for children ≥ 6 years.

Exclusion criteria

- evidence of lacunar or follicular angina;
- > two episodes of tonsillitis within the last 12 months;
- mandatory indication for therapy with antibiotics (e.g., abscess, septic tonsillitis);
- treatment with antibiotics within 4 months prior to study inclusion;
- increased haemorrhagic diathesis, chronic diseases (e.g., severe heart, kidne;
- close contact with SARS-COV-2 infected individuals in the last 10 days;
- known or suspected hypersensitivity to study medication;
- concomitant treatment potentially influencing study outcome;
- participation in another clinical study within the last 3 months.

Treatments

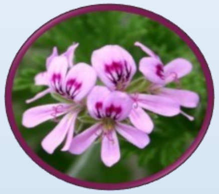

A 6-day study with administration of an extract of **Pelargonium sidoides** in pediatric patients with acute non-streptococcal tonsillopharyngitis showed a **clinically relevant decrease of disease symptomps** significantly superior to placebo[1].

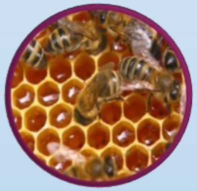

Zinc reduces the **average duration** of the **common cold** in healthy people assuming Zinc within 24 hours of onset symptoms[2].

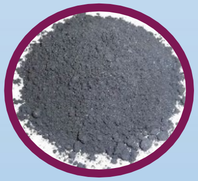

Propolis is administered as an add-on theapy in non-streptococcal pharyngitis[3].

1. *Bereznoy VV et al., Efficacy of extract of Pelargonium sidoides in children with acute non-group A beta-hemolytic streptococcus tonsillopharyngitis: a randomized, double-blind, placebo-controlled trial. Altern Ther Health Med 2003 Oct;*
2. *Singh M, Das RR. Zinc for the common cold. Cochrane Database Syst Rev 2011 Feb 16;*
3. *Di Pierro F, Zanvit A, Colombo M. Role of a proprietary propolis-based product on the wait-and-see approach in acute otitis media and in preventing evolution to tracheitis, bronchitis, or rhinosinusitis from nonstreptococcal pharyngitis. Int J Gen Med 2016.*

Treatments

Control group: Standard of Care (SoC).

vs

Interventional group: Dietary Supplement (DSPP) + SoC

- 5ml x 3/day orally for children < 6 years for 6 days
- 10ml x 3/day orally for children ≥ 6 years for 6 days

SoC
Product for nasopharyngeal liberation.
benzydamine hydrochloride
Paracetamol (or Ibuprofen)

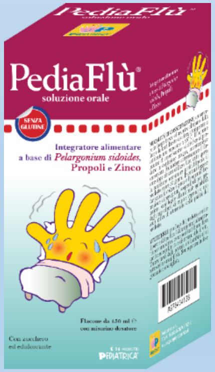

DSPP
***Pelargonium sidoides*,**
Propolis (PropolNext® PLUS)
and ***Zinc***

Primary Outcomes

TSS (Tonsillitis Severity Score): change of TSS from baseline to final visit between groups

Number of treatment failures: rescue medicine (Ibuprofen or dosage of over 30 mg/kg/dose of Paracetamol) compared in the two groups

AE/SAE: incidence of Adverse Events/Serious Adverse Events

Secondary Outcomes

PGAE (Patient Global Assessment of Efficacy)

IGAE (Investigator Global Assessment of Efficacy)

IGAS (Investigator Global Assessment of Safety)

Investigational Product compliance

Thank you for your time

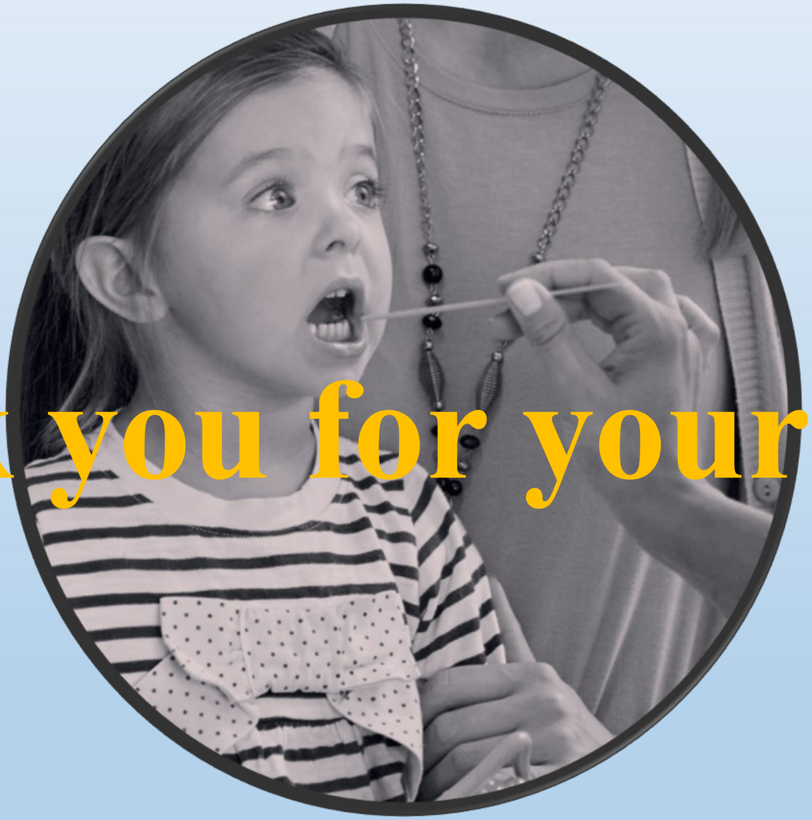

Supplement: Multimedia Appendix 1 [file resprot_v13i1e53703_app1.pdf]
