# Supplementary material for: The Effects of a Dietary Supplement (PediaFlù) Plus Standard of Care in Children With Acute Tonsillopharyngitis/Rhinopharyngitis: Protocol for a Randomized Controlled Trial
Source: JMIR Res Protoc. 2024 May 31;13:e53703. doi: 10.2196/53703 (PMC11179036; doi:10.2196/53703)
Supplement: Multimedia Appendix 2 [file resprot_v13i1e53703_app2.pdf]

CABINET MEDICAL MEDICINĂ DE FAMILIE DR. MORARIU BORDEA
Bld. Liviu Rebreanu, nr.28, Timișoara, România

Decizia Comisiei Locale de Etică pentru cercetare științifică a

CABINETULUI MEDICAL MEDICINĂ DE FAMILIE DR. MORARIU BORDEA

Ca răspuns la adresa Dumneavoastră, vă comunicam avizarea din punct de vedere etic al studiului clinic intitulat:

"Studiu deschis, randomziat, cu două brațe, controlat, pentru evaluarea eficacității și siguranței PediaFlù® (supliment alimentar) împreună cu terapie standard la copiii cu faringită acută/rinofaringită versus terapie standard"

Număr de protocol: **OPPED/0120/FS,** Versiune finală **3.0 din 16 Mar. 2021**

SIUIInvestigator Principal: ***Dr. MORARIU BORDEA***

Comisia Locală de Etică pentru cercetare științifică a **CABINETULUI MEDICAL MEDICINĂ DE FAMILIE DR. MORARIU BORDEA** funcționează în conformitate cu prevederile art. 167 din Legea nr. 95/2006, art. 28, cap. VIII din ordinul 904/2006, Directivei 2001/20/EC a Parlamentului European și a consiliului din 4 Aprilie 2001 și cu Declarația de la Helsinki –ediția 64th emisă de OMS la Fortalezza, Brazilia, Octombrie 2013.

În urma analizei documentelor transmise, Comisia de Etică avizează **favorabil** desfășurarea studiului clinic sus-mentionat.

Cu stimă,

Dr. Trut Marinela - Președinte Comisia de Etică ____________

Dr. Dragomir Adriana - Membru Comisia de Etică ____________

Szekeres Daniela - Membru Comisia de Etică ____________

Cabau Daniel - Membru Comisia de Etică ____________

Hodor Cristina - Membru Comisia de Etică ____________

Timișoara,

Data 27.04.2021

C.M. Dr. Herțeg Dorina
Calea Sever Bocu, nr. 33, 300288 Timișoara, România

Decizia Comisiei Locale de Etică pentru cercetare științifică a

C.M. Dr. Herțeg Dorina

Ca răspuns la adresa Dumneavoastră, vă comunicăm avizarea din punct de vedere etic al studiului clinic intitulat:

"Studiu deschis, randomziat, cu două brațe, controlat, pentru evaluarea eficacității și siguranței PediaFlù® (supliment alimentar) împreună cu terapie standard la copiii cu faringită acută/rinofaringită versus terapie standard"

Număr de protocol: **OPPED/0120/FS,** Versiune finală **3.0 din 16 Mar 2021**

Investigator Principal: *Dr. Herțeg Dorina*

Comisia Locală de Etică pentru cercetare științifică a **C.M. Dr. Herțeg Dorina** funcționează în conformitate cu prevederile art. 167 din Legea nr. 95/2006, art. 28, cap. VIII din ordinul 904/2006, Directivei 2001/20/EC a Parlamentului European și a consiliului din 4 Aprilie 2001 și cu Declarația de la Helsinki –editia 64th emisă de OMS la Fortalezza, Brazilia, Octombrie 2013.

În urma analizei documentelor transmise, Comisia de Etică avizează **favorabil** desfășurarea studiului clinic sus-mentionat.

Cu stimă,

Dr. Crețu Zanfira - Președinte Comisia de Etică ____________

Dr. CREȚU ZANFIRA
medic primar
medicină generală pediatrie
cod [illegible]

Dr. Cernazan Glavan Anda - Membru Comisia de Etică ____________

Dr. CERNĂZANU-GLĂVAN MIHAELA
medic primar
medicină de familie
Cod: C10624

Radu Florina - Membru Comisia de Etică ____________

Krizja Alexandra - Membru Comisia de Etică ____________

Reizner Kariana - Membru Comisia de Etică ____________

Timișoara, Data 27.04.2021

CM Dr. Matei Cristian-Radu
Oțelu Roșu, Strada Republicii nr. 14-16, România

Decizia Comisiei Locale de Etică pentru cercetare științifică a

CM Dr. Matei Cristian-Radu

Ca răspuns la adresa Dumneavoastră, vă comunicăm avizarea din punct de vedere etic al studiului clinic intitulat:

"Studiu deschis, randomziat, cu două brațe, controlat, pentru evaluarea eficacității și siguranței PediaFlù® (supliment alimentar) împreună cu terapie standard la copiii cu faringită acută/rinofaringită versus terapie standard"

Număr de protocol: **OPPED/0120/FS,** Versiune finală **3.0 din 16 Mar 2021**

Investigator Principal: ***Dr. Matei Cristian Radu***

Comisia Locală de Etică pentru cercetare științifică a **CM Dr. Matei Cristian-Radu** funcționează în conformitate cu prevederile art. 167 din Legea nr. 95/2006, art. 28, cap. VIII din ordinul 904/2006, Directivei 2001/20/EC a Parlamentului European și a consiliului din 4 Aprilie 2001 și cu Declarația de la Helsinki –editia 64th emisă de OMS la Fortalezza, Brazilia, Octombrie 2013.

În urma analizei documentelor transmise, Comisia de Etică avizează **favorabil** desfășurarea studiului clinic sus-mentionat.

Cu stimă,

- Dr. Popovici Ion - Președinte Comisia de Etică ____________
- Maftei Mariana - Membru Comisia de Etică ____________
- Hertila Nicoleta - Membru Comisia de Etică ____________
- Megan Vasile - Membru Comisia de Etică ____________
- Tamaș Mirabela - Membru Comisia de Etică ____________

Timișoara,

Data 23.04.2021
